# Prevalence and Risk Factors of Elevated Alanine Aminotransferase (ALT) in 2382 Treatment‐naïve HBV/HDV Co‐Infected Patients

**DOI:** 10.1111/liv.70559

**Published:** 2026-02-26

**Authors:** Habiba Kamal, Ganbolor Jargalsaikhan, Sanjaasuren Enkhtaivan, Karin Lindahl, Hannes Hagström, Daniel Bruce, Michael Ingre, Bekhbold Dashtserenx, Oyungerel Lkhagva‐Ochir, Tuvshinjargal Ulziibadrakh, Andreas Bungert, Heiner Wedemeyer, Naranjargal B. Dashdorj, Soo Aleman

**Affiliations:** ^1^ Department of Infectious Diseases Karolinska University Hospital Stockholm Sweden; ^2^ Department of Medicine Huddinge Karolinska Institutet Stockholm Sweden; ^3^ The Liver Center Ulaanbaatar Mongolia; ^4^ ONOM Foundation Ulaanbaatar Mongolia; ^5^ Unit of Hepatology, Department of Upper GI Diseases Karolinska University Hospital Stockholm Sweden; ^6^ Cytel Statistical Consultancy Stockholm Sweden; ^7^ Department of Gastroenterology, Hepatology, Infectious Diseases and Endocrinology Hannover Medical School Hannover Germany

**Keywords:** alanine aminotransferase, cirrhosis, HBV, hepatitis D, immunotolerance

## Abstract

**Background:**

Chronic hepatitis D (CHD) causes severe chronic hepatitis. Knowledge is limited about factors correlating with ALT in treatment‐naïve patients with CHD. This study analysed the pattern and determinants of ALT elevation in a large cohort of patients with CHD, including young adults, compared to propensity score‐matched (PSM) patients with chronic hepatitis B (CHB).

**Methods:**

We identified 2382 treatment‐naïve HBsAg+ adults with CHD (HDV RNA positive) and 1553 with CHB attending a liver center in Mongolia during 2015–2023. The correlation between ALT levels, virological, biochemical, and fibrosis parameters was assessed using Spearman coefficient (rho). Logistic regression analysis was used to identify determinants of elevated ALT in 1371 PSM pairs with CHD and CHB matched on age, sex, metabolic factors, and date of initial test.

**Results:**

In CHD, 78.5% of patients had ALT elevation, with the highest prevalence in the 18–20 years group (*n* = 219, 84.5%). This age group displayed 8.2‐adjusted odds ratio (aOR) for elevated ALT, 2.7‐aOR for elevated GGT, and 4.5‐aOR of cirrhosis than matched CHB group (all *p* < 0.05). In CHD, ALT correlated weakly with HDV RNA (rho = 0.23) and liver stiffness (rho = 0.37), moderately with GGT (rho = 0.48), while showed no correlation with HBV DNA or HBsAg. Independent factors for elevated ALT were age < 30 years, elevated GGT and HDV RNA levels.

**Conclusions:**

In this large cohort of Asian patients, an earlier and more severe inflammatory process could be demonstrated in CHD compared to CHB regardless of liver cirrhosis. Longitudinal studies are warranted to risk‐stratify and prioritise patients for therapies.

## Key Points

- The prevalence and determinants of alanine aminotransferase (ALT) elevation were studied in a large cohort of 2382 treatment‐naïve patients with chronic hepatitis D (CHD) compared to 1553 with HBV mono‐infection.
- Young patients (< 30 years of age) with CHD showed more prevalent ALT elevation and HBeAg+ than propensity‐score matched peers with HBV mono‐infection.
- ALT elevation in CHD was independently associated with younger age, elevated GGT and HDV RNA levels.
- ALT elevation in young adults with CHD and HBeAg+ suggested a more aggressive and prolonged phase of immune response.

**Abbreviations**
ALT: alanine aminotransferase
CHD: chronic hepatitis D
MRF: metabolic risk factor

## 1 Introduction

Chronic hepatitis D (CHD) is associated with an aggressive liver disease course [1]. HBV/HDV co‐infection affects nearly 9 to 19 million individuals worldwide [2]. The prevalence is highest in Mongolia, Southern Asian countries such as Pakistan as well as the Amazon basin and West Africa, while generally low (< 0.2%) in Western countries, where the majority of patients consist of migrants from endemic regions [2, 3]. CHD is classified as a rare disease in the European Union, with orphan designation when developing new HDV drugs [4]. At diagnosis, 30%–50% of patients with CHD present with advanced liver fibrosis, at a mean age of 40 years [5, 6].

Elevated alanine aminotransferase (ALT) level reflects hepatic necro‐inflammatory process, and correlates with liver‐related outcomes such as cirrhosis and hepatocellular carcinoma (HCC) [7]. Nevertheless, ALT normalisation is an accepted surrogate marker for treatment response and improved clinical outcomes in HDV drug trials [8, 9]. Knowledge about ALT levels is therefore crucial to understand the pathological process and to guide the management of CHD, particularly in the light of new anti‐HDV agents in development.

While ALT elevation is frequent in CHD, data on its prevalence, severity, and association with patients' characteristics and other factors across the natural course of untreated CHD remain scarce [6, 10, 11]. Particularly, data on young adults with CHD compared to chronic hepatitis B monoinfection (CHB) are absent. Studies on CHD have been constrained by small sample size, inclusion of both treatment‐naïve and experienced patients, usually from tertiary care settings, possibly reducing statistical power and selection bias of more advanced CHD [6, 10, 11].

To address this knowledge gap, we analysed the prevalence and factors correlating with ALT elevation in CHD patients, particularly young adults, and compared them to propensity score‐matched (PSM) CHB patients. This analysis was enabled using a large cohort of treatment‐naïve patients with CHD and CHB from a liver center in Mongolia, a country with a substantial burden of viral hepatitis [12, 13].

## 2 Patients and Methods

### 2.1 Study Population

**FIGURE 1:**
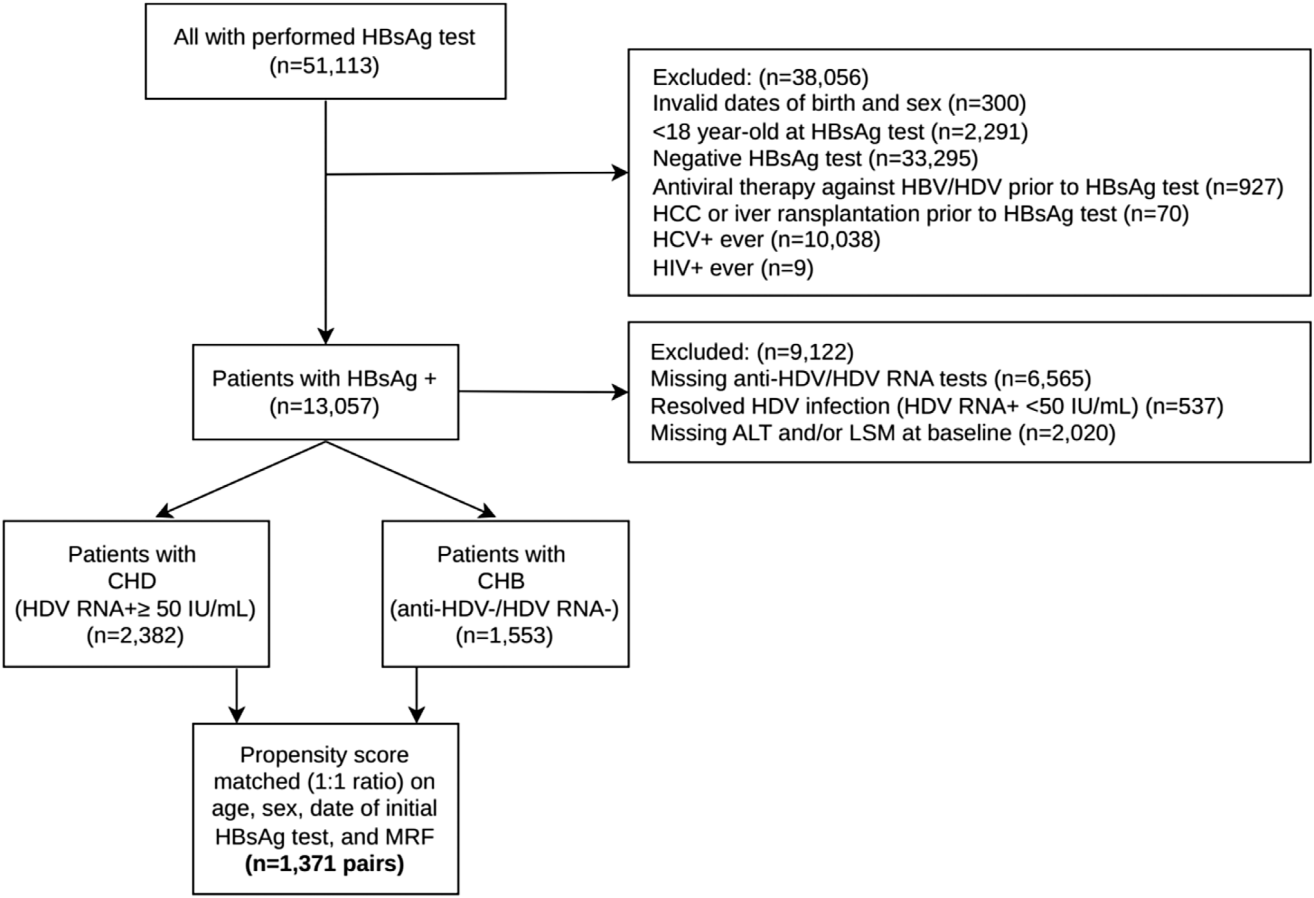
Study flowchart. All laboratory and virological parameters within (< 90 days) from baseline. ALT, alanine aminotransferase; CHB, chronic hepatitis B; CHD, chronic hepatitis D; HCC, hepatocellular carcinoma; HCV, hepatitis C virus; HIV, human immunodeficiency virus; LSM, liver stiffness measurement by transient elastography.

We identified 51 113 adult individuals (≥ 18 years) with HBsAg testing at the Liver Center, Ulaanbaatar in Mongolia, between from 1st January 2015 to 31st December 2023 (Figure 1). Data were retrieved from an electronic database, comprising medical and investigations records. The Liver Center provides outpatient care for patients with liver diseases, and also screens individuals for viral hepatitis upon self‐referral. After exclusions (*n* = 38 056), patients with HBsAg positive without prior record of anti‐HBV or anti‐HDV therapies were categorised on their virological parameters and available alanine aminotransferase (ALT) and liver stiffness measurement (LSM) into two cohorts: (1) HBV/HDV co‐infection or CHD, if at least one serum HDV RNA level ≥ 50 IU/mL (*n* = 2382), and (2) mono‐HBV infection or CHB, with at least one negative anti‐HDV and/or HDV RNA test, and no record of anti‐HDV or HDV RNA positivity (*n* = 1553).

The term HBV/HDV co‐infection used in this study interchangeably with CHD, and do not refer to the type of infection as simultaneous or superinfection.

All data were anonymized and transferred to Karolinska University Hospital and Karolinska Institutet, Sweden for analysis. Ethical approval was granted by the local ethics review board at the Liver center in Mongolia and by the Ethical Review Authority of Sweden. The study was funded by an ALF grant from Region Stockholm, Sweden.

### 2.2 Demographic, Biochemical Test and Fibrosis Staging

The baseline date was the first positive HBsAg test date. Demographic data, including date of birth and sex, and medical data such as body mass index (BMI), antiviral treatments and liver‐related diagnoses as liver cirrhosis, hepatocellular carcinoma, decompensation events (ascites, variceal bleeding, or encephalopathy) and liver transplantation during the study period of 1st January 2015 to 31st December 2023 were retrieved.

Body mass index (BMI) was calculated as weight in kilogram divided by height in meters squared (kg/m^2^). The following cut‐offs were used for BMI categories in accordance with Asian population standards: underweight (< 18.5 kg/m^2^), normal weight (18.5–22.9 kg/m^2^), overweight (23.0–27.4 kg/m^2^) and obesity (≥ 27.5 kg/m^2^) [14].

The upper limit of normal (ULN) for biochemical tests, as per local laboratory routines, was defined as follows: ALT of 41 IU/L for men and 31 IU/L for women, AST of 35 IU/L for men and 31 IU/L for women, GGT of 55 IU/L for men and 38 IU/L for women, serum triglyceride of 1.7 mmol/L, and serum low‐density lipoproteins of 2.6 mmol/L. The lower limit of normal (LLN) for serum high density lipoproteins was 1.0 mmol/L for men and 1.3 mmol/L for women.

Metabolic risk factors were defined as the presence of at least one of the following conditions: overweight or obesity, diabetes mellitus (either a documented diagnosis or fasting serum glucose ≥ 7.0 mmol/L on two occasions), dyslipidemia, hypertension, and/or diagnosis of fatty liver disease [15]. Events of HCC, decompensation events (defined as ascites, variceal bleeding, or encephalopathy), or liver transplantation were retrieved from medical records, as well as from ultrasound or gastroscopy investigation records where appropriate.

For CHD, the LSM cut‐offs used to define advanced/F3 fibrosis was ≥ 10.0–< 15.0 kPa, while ≥ 15.0 kPa for F4 fibrosis [16]. The corresponding LSM cut‐off levels for CHB were ≥ 9.0–< 12.5 kPa for F3 and ≥ 12.5 kPa for F4 fibrosis [17]. LSM values with a success rate of ≥ 90% and the interquartile range (IQR) of ≤ 30% were considered reliable and used [17]. Since LSM levels may be affected by elevated transaminases due to liver necroinflammation [18], analyses using platelets count as an alternative marker for advanced fibrosis/cirrhosis were performed. For cirrhosis diagnosis, criteria of either a LSM value of ≥ 15.0 for CHD or ≥ 12.5 kPa for CHB, or platelet counts of < 150 × 10^9^ cells/L were utilized [19].

### 2.3 Serological and Virological Tests

HBsAg was tested with either rapid diagnostic test (CTK Co.LtD., USA), or quantitative HBsAg test (Sysmex Co.Ltd., Japan) at baseline. Anti‐HCV and anti‐HIV were qualitatively tested by rapid diagnostic test (CTK Co.LtD., USA). Quantitative HBsAg was estimated by HISCL‐5000 fully automated chemiluminescence analyser (Sysmex Co.Ltd., Japan) and for HBeAg status (CLIA, Sysmex, Japan, lower level of detection (LOD) of 1 COI). HCV RNA was quantified using real time reverse transcriptase polymerase chain reaction with LOD of 10 IU/mL (GeneXpert, Cepheid, USA). Anti‐HDV was analysed using the Wantai HDV‐IgG ELISA (Wantai Biopharmaceutical Co. Ltd., China), with a lower limit of detection (LoD) of < 1 COI. HDV RNA levels were quantified, using a Bioactiva Diagnostica extraction kit (Bioactiva Diagnostica, Germany) and a Bio‐Rad amplification kit (Bio‐Rad Laboratories, USA) with a lower limit of quantification (LoQ) of < 10 IU/mL. HBV DNA levels were analysed using the GeneXpert system (Cepheid, USA), with an LoQ of < 10 IU/mL.

### 2.4 Statistical Analysis

Continuous variables were compared using Student‐t or the Mann–Whitney *U* tests, while categorical variables were compared using the Chi‐Square test or Fisher's exact test. For correlation analyses, parameters within ±21 nearest to baseline (HBsAg test) with no prior HCC diagnosis, initiation of antiviral treatment, or liver transplantation were considered, while for baseline analyses, variables were used within 90 days of baseline. Correlations between paired parameters were assessed using Spearman's correlation coefficient (rho). Coefficient values were interpreted as follows: very strong (0.80–1.00), strong (0.60 to < 0.80), moderate (0.40 to < 0.60), weak (0.20 to < 0.40), and very weak (> 0.0 to < 0.20) [20]. Scatter plots with overlay smooth lines were used to visualise univariable correlations, representing trends in data. Univariable and multivariable logistic regression analyses were conducted to identify parameters significantly associated with ALT elevation (> ULN) defined as above [16]. Results were presented as odds ratio (OR) and 95% confidence intervals (CI). Propensity scores matching (PSM) were constructed using logistic regression model with CHD as the outcome, and variables age, sex, MRF and date of HBsAg test as covariates. The model estimated the probability of being CHD per each patient, and the propensity scores were used in 1:1 nearest neighbour matching for age and date of HBsAg and exact matching for sex and MRF to balance the groups. After matching, the standardised mean differences (SMD) between CHB and CHD were assessed. All analyses were similarly conducted on the matched pairs.

Statistical significance was considered when *p*‐values < 0.05. Data analyses were executed using R Studio version 3 and IBM SPSS Statistics, version 28.0.1.1.

## 3 Results

### 3.1 Baseline Characteristics of Patients With CHD by Age Groups

**FIGURE 2:**
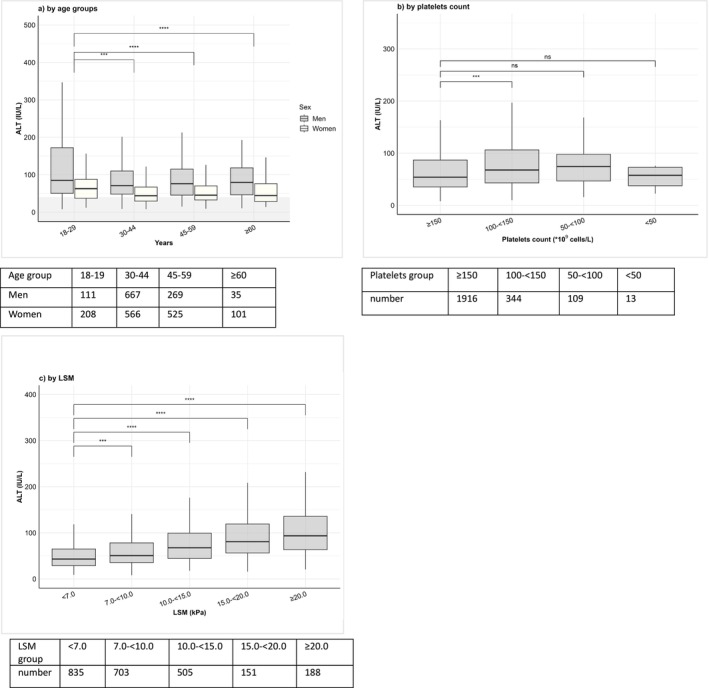
Box plot illustrating ALT levels, subgrouped by (a) age in men and women, (b) platelet counts, and (c) by LSM measurements in treatment‐naïve patients with chronic hepatitis D. ALT levels on the y‐axis, and age categories, liver stiffness, and platelet count subgroups are shown on the x‐axis. Median ALT values are represented by interior bars, with upper and lower borders representing the 25th and 75th quartiles, with whiskers of minimum and maximum values. The shaded area marks the upper limit of normal ALT for men (< 41 IU/L) and women (< 31 IU/L), respectively. Significance was evaluated by the *t*‐test. The earliest laboratory values within 90 days of first HBsAg, anti‐HDV and HDV RNA positive tests without exposure to anti‐HDV or anti‐HBV therapies were considered. ALT, alanine aminotransferase; LSM, liver stiffness measurement.

As shown in Table 1, patients aged 18–29 years‐old constituted 9.2% of the cohort, men were less prevalent in older age groups compared to women (50.7% in 18–29 years vs. 25.7% in ≥ 60 years, *p* < 0.001). Group of 18–29 years had the highest median ALT 69.4 (44.2–122.8) IU/L, with 15.5% having normal level (*p* < 0.001), also shown in Figure 2a. To the contrast, GGT level increased with age with elevated levels at 35.0% among 18–29 years reaching 43.7% in older age groups (*p* < 0.001). Young age group also had the highest prevalence of HBeAg+ at 51.4%, higher HBsAg level at 3.9 log_10_ IU/mL and lower median HBV DNA level at 2.1 log_10_ IU/mL compared to older age groups (*p* < 0.001). Of note, HDV RNA level was similar across all age groups (*p* = 0.2) (Table 1 and Figure 2).

**TABLE 1:** Baseline characteristics of 2382 patients with chronic hepatitis D (CHD) by age groups.

Parameters	*N*	All	18–29	30–44	45–59	≥ 60	*p*
Number		2382	219 (9.2)	1233 (51.8)	794 (33.3)	136 (5.7)	
Age at first HBsAg test, years, median (IQR)	2382	41.2 (34.3, 50.7)	28.0 (26.6, 29.2)	36.9 (33.5, 40.6)	51.6 (48.2, 55.3)	62.9 (61.1, 66.0)	< 0.001
Sex	2382						< 0.001
Female		1300 (54.6)	108 (49.3)	566 (45.9)	525 (66.1)	101 (74.3)	
Male		1082 (45.4)	111 (50.7)	667 (54.1)	269 (33.9)	35 (25.7)	
BMI, median (IQR)	938	26.4 (23.4, 29.7)	24.1 (21.3, 28.4)	26.3 (23.2, 29.8)	27.2 (24.5, 30.2)	24.7 (22.4, 28.0)	< 0.001
ALT, IU/L, median (IQR)	2382	56.6 (36.5, 92.0)	69.4 (44.2, 122.8)	57.5 (36.9, 92.1)	52.0 (34.9, 86.2)	48.6 (30.6, 81.4)	< 0.001
**ALT level categories adjusted for sex**	2382						
Normal ALT level, < 1ULN		511 (21.5)	34 (15.5)	273 (22.1)	168 (21.2)	36 (26.5)	
≥ × 1ULN–< 2 × ULN		983 (41.3)	71 (32.4)	523 (42.4)	342 (43.1)	47 (34.6)	
≥ 2 × ULN–< 5 × ULN		721 (30.3)	90 (41.1)	349 (28.3)	237 (29.8)	45 (33.1)	
≥ 5 × ULN–< 10 × ULN		141 (5.9)	18 (8.2)	72 (5.8)	43 (5.4)	8 (5.9)	
≥ 10 × ULN		26 (1.1)	6 (2.7)	16 (1.3)	4 (0.5)	0	
AST, IU/L, median (IQR)	2375	42.1 (30.4, 63.9)	46.9 (33.8, 74.7)	40.0 (29.5, 61.5)	44.3 (31.6, 65.1)	45.6 (30.0, 68.4)	< 0.001
GGT, IU/L, median (IQR)	1841	39.6 (25.4, 64.8)	36.2 (21.9, 56.6)	38.7 (25.1, 63.2)	42.4 (26.5, 73.3)	38.0 (27.1, 56.7)	0.008
Elevated GGT, ≥ 55 IU/L in male, ≥ 38 IU/L in female	1841	774 (42.0)	55 (35.0)	377 (38.5)	297 (49.3)	45 (43.7)	< 0.001
Albumin, g/L, median (IQR)	2082	42.0 (39.6, 44.2)	43.3 (40.7, 45.2)	42.3 (40.1, 44.4)	41.2 (38.8, 43.5)	41.4 (38.6, 43.6)	< 0.001
Total bilirubin, μmol/L, median (IQR)	2219	12.9 (9.6, 16.8)	12.7 (9.0, 15.4)	13.0 (9.6, 17.2)	12.9 (9.7, 16.5)	13.4 (10.5, 17.4)	0.2
Conjugated bilirubin, μmol/L, median (IQR)	1486	4.6 (3.6, 6.1)	5.0 (3.5, 6.4)	4.6 (3.5, 6.1)	4.6 (3.6, 6.0)	4.9 (3.8, 6.3)	0.5
Platelets count, 10^9^ cells/L, median (IQR)	2382	199.0 (159.8, 236.0)	214.0 (185.5, 251.5)	204.0 (168.0, 240.2)	184.0 (148.1, 225.5)	184.0 (154.9, 231.6)	< 0.001
**Virological parameters**							
HBeAg, positive	560	232 (41.4)	37 (51.4)	132 (42.2)	55 (36.4)	8 (33.3)	0.2
*HBsAg (IU/mL)*							
qHBsAg log_10_, median (IQR)	1930	3.8 (3.4, 4.1)	3.9 (3.5, 4.1)	3.9 (3.5, 4.1)	3.8 (3.4, 4.1)	3.7 (3.2, 4.0)	< 0.001
qHBsAg < 3 log_10_		244 (12.6)	23 (14.5)	108 (10.7)	89 (13.7)	24 (22.0)	0.004
qHBsAg 3– < 4 log_10_		1006 (52.1)	76 (47.8)	520 (51.5)	356 (54.6)	54 (49.5)	
qHBsAg ≥ 4 log_10_		680 (35.2)	60 (37.7)	382 (37.8)	207 (31.7)	31 (28.4)	
*HDV RNA (IU/mL)*							
qHDV RNA log_10_, median (IQR)	2382	5.3 (4.3, 6.1)	5.5 (4.3, 6.6)	5.3 (4.2, 6.2)	5.3 (4.3, 6.0)	5.3 (4.3, 6.1)	0.2
qHDV RNA < 3 log_10_		292 (12.3)	34 (15.5)	161 (13.1)	78 (9.8)	19 (14.0)	0.10
qHDV RNA 3– < 5 log_10_		683 (28.7)	53 (24.2)	345 (28.0)	249 (31.4)	36 (26.5)	
qHDV RNA ≥ 5 log_10_		1407 (59.1)	132 (60.3)	727 (59.0)	467 (58.8)	81 (59.6)	
qHBV DNA log_10_, median (IQR)	1732	2.4 (1.5, 3.2)	2.1 (1.5, 3.2)	2.2 (1.4, 3.2)	2.4 (1.6, 3.2)	3.0 (2.4, 3.5)	< 0.001
LSM, median (IQR)	2382	8.3 (6.1, 11.7)	7.6 (5.8, 10.4)	7.9 (6.1, 11.0)	8.8 (6.7, 12.7)	8.9 (6.8, 13.5)	< 0.001
LSM < 7.5 kPa	2382	987 (41.4)	104 (47.5)	561 (45.5)	275 (34.6)	47 (34.6)	< 0.001
LSM ≥ 10.0 kPa	2382	835 (35.1)	65 (29.7)	390 (31.6)	320 (40.3)	60 (44.1)	< 0.001
MRF	2382	845 (35.5)	55 (25.1)	414 (33.6)	336 (42.3)	40 (29.4)	< 0.001
Cirrhosis^a^	2382	612 (25.7)	36 (16.4)	269 (21.8)	261 (32.9)	46 (33.8)	< 0.001

*Note:* Number and proportions are presented by column; parameters are presented as *n* (%) unless stated otherwise.

Abbreviations: ALT, alanine aminotransferase; AST, aspartate aminotransferase; BMI, body mass index; GGT, gamma glutamyl transferase; IQR, 25th–75th interquartile; LSM, liver stiffness measurements; MRF, metabolic risk factor; q, quantitative; sd, standard deviation; ULN, upper limit of normal.

^a^ Cirrhosis = defined as liver stiffness measurement ≥ 15.0 kPa or platelets counts < 150 × 10^9^ cells/L.

### 3.2 Factors Associated With Elevated ALT Levels in CHD

As shown in Table 2, younger age < 30 year‐old, male sex, higher BMI, lower platelets count, AST, GGT, HBsAg, HDV RNA, and record of cirrhosis were all significantly associated with elevated ALT in CHD in univariable analysis. In multivariable analysis, age < 30 year‐old carrying aOR = 2.16 (1.26–3.92), elevated GGT level; aOR = 4.64 (3.35–6.54), and HDV RNA log_10_ IU/mL; aOR = 1.48 (1.36–1.62) remained significant after adjusting for age, sex, HBsAg, HDV RNA, and cirrhosis.

**TABLE 2:** Parameters associated with elevated alanine aminotransferase (ALT) above upper limit of normal (ULN) in chronic hepatitis D (CHD).

Predictor	Factor	Univariable				Multivariable			
		OR	LCI	UCI	*p*	aOR	LCI	UCI	*p*
Age at initial test	Continuous	0.99	0.98	1.00	0.12				
Age < 30 years of age	Yes, vs. no	1.54	1.07	2.29	0.03	2.16	1.26	3.92	< 0.001
Sex (male)	Yes, vs. no	1.51	1.23	1.84	< 0.001	1.22	0.94	1.60	0.38
BMI, kg/m^2^	Continuous	1.08	1.04	1.12	< 0.001				
Platelet count, ×10^9^ cells/L	Continuous	1.00	0.99	1.00	< 0.001				
Platelet count, < 150 × 10^9^ cells/L	Yes, vs. no	1.87	1.42	2.50	< 0.001	1.34	0.91	2.02	0.15
AST, IU/L	Continuous	1.20	1.18	1.22	< 0.001				
GGT, IU/L	Continuous	1.02	1.02	1.03	< 0.001				
Elevated GGT>ULN, IU/L	Yes, vs. no	4.96	3.73	6.70	< 0.001	4.64	3.35	6.54	< 0.001
HBeAg positive	Yes, vs. no	1.32	0.85	2.08	0.22				
qHBsAg log_10_, IU/mL	Continuous	1.62	1.40	1.88	< 0.001	1.04	0.78	1.40	0.76
qHBV DNA log_10_, IU/mL	Continuous	1.02	0.93	1.11	0.68				
qHDV RNA log_10_, IU/mL	Continuous	1.45	1.36	1.55	< 0.001	1.48	1.36	1.62	< 0.001
LSM, kPa	Continuous	1.10	1.08	1.13	< 0.001				
Cirrhosis^a^,^b^	Yes, vs. no	2.22	1.72	2.90	< 0.001	1.70	0.87	3.70	0.14
Diabetes	Yes, vs. no	1.70	0.90	3.53	0.13				
MRF	Yes, vs. no	1.16	0.94	1.43	0.17				

*Note:* Crude odds ratio (OR) and adjusted (aOR) with 95% confidence interval, CI are derived through univariable and multivariable logistic regression analysis. In men the ULN for ALT was 41 IU/L, 35 IU/L for AST, 55 IU/L for GGT and in women 31 IU/L, 31 IU/L and 38 IU/L, respectively. Multivariable model adjusted for age < 30, sex, platelets count or cirrhosis, HBsAg log_10_, and HDV RNA log_10_.

Abbreviations: ALT, alanine aminotransferase; AST, aspartate aminotransferase; BMI, body mass index; CHD, chronic hepatitis D; GGT, gamma glutamyl transferase; LSM, liver stiffness measurements; MRF, metabolic risk factor; q, quantitative; ULN, upper limit of normal.

^a^ Cirrhosis = defined as liver stiffness measurement ≥ 15.0 for CHD and ≥ 12.5 for CHB or platelets counts < 150 × 10^9^ cells/L.

^b^ Tested in the multivariable model without platelets count.

### 3.3 Baseline Differences Between Patients With CHD and CHB in All Cohort and in PSM Matched

Compared to CHB, patients with CHD were older (mean age 41.2 vs. 34.3 years old, *p* < 0.001), with more prevalent women (54.6% vs. 45.6%, *p* < 0.001) (Table S1).

In CHD, median ALT value was 56.6 (36.5–92.0) IU/L with normal level in 21.5% vs. 62.3% in CHB (*p* < 0.001). CHD showed higher AST, GGT, bilirubin, and lower albumin, and platelets count (all *p* < 0.05). They showed also higher HBsAg and lower HBV DNA levels, more prevalent MRF (35.5% vs. 32.3%) and more cirrhosis record (25.7% vs. 7.9%, *p* < 0.001).

*We performed a* propensity‐score matching (on age, sex, MRF and date for HBsAg test) which yielded 1371 matched pairs of CHD and CHB. The SMD for propensity score (distance) was 0.29, for sex was 0.0001, for age 0.03, for date of HBsAg was −0.67, and for MRF was 0.001. All SMDs were < 0.1 except for date of HBsAg test, indicating good balance across the matched covariates (results not tabulated). Similar to main cohorts, PSM matched patients with CHD showed higher ALT, AST, GGT, bilirubin, LSM, and higher prevalence of cirrhosis as shown in (Table S1).

To delineate the effect of CHD vs. CHB across age groups, the association of CHD with baseline parameters is presented in (Table S2). Adjusting for age, sex, ALT, HBeAg, HBV DNA, HBsAg, MRF, and cirrhosis; age group 18–29 years with CHD demonstrated 6.53‐aOR (95% CI 1.84–28.40) of elevated ALT, 11.0‐aOR (95% CI 1.79–219.0) of HBeAg+, 22.3‐aOR (95% CI 3.56–451.20) of low HBV DNA and similar high HBsAg levels and cirrhosis risks compared to the same age group with CHB. Patients with CHD had overall higher odds of HBeAg+ compared to CHB with aOR of 3.57 (95% CI 2.10–6.27), also shown in age subgroups. Patients with CHD showed higher odds of HBV DNA < 2000 IU/mL, more pronounced in the younger age group; aOR = 22.30, and in those aged ≥ 60 years aOR = 4.36.

### 3.4 Pair‐Wise Correlations of ALT With Biochemical and Virological Parameters in CHD

ALT levels were higher in platelets count 50–< 100, 100–< 150, and declined in those with < 50, while correlated positively with LSM subgroups (Figure 2b,c).

**FIGURE 3:**
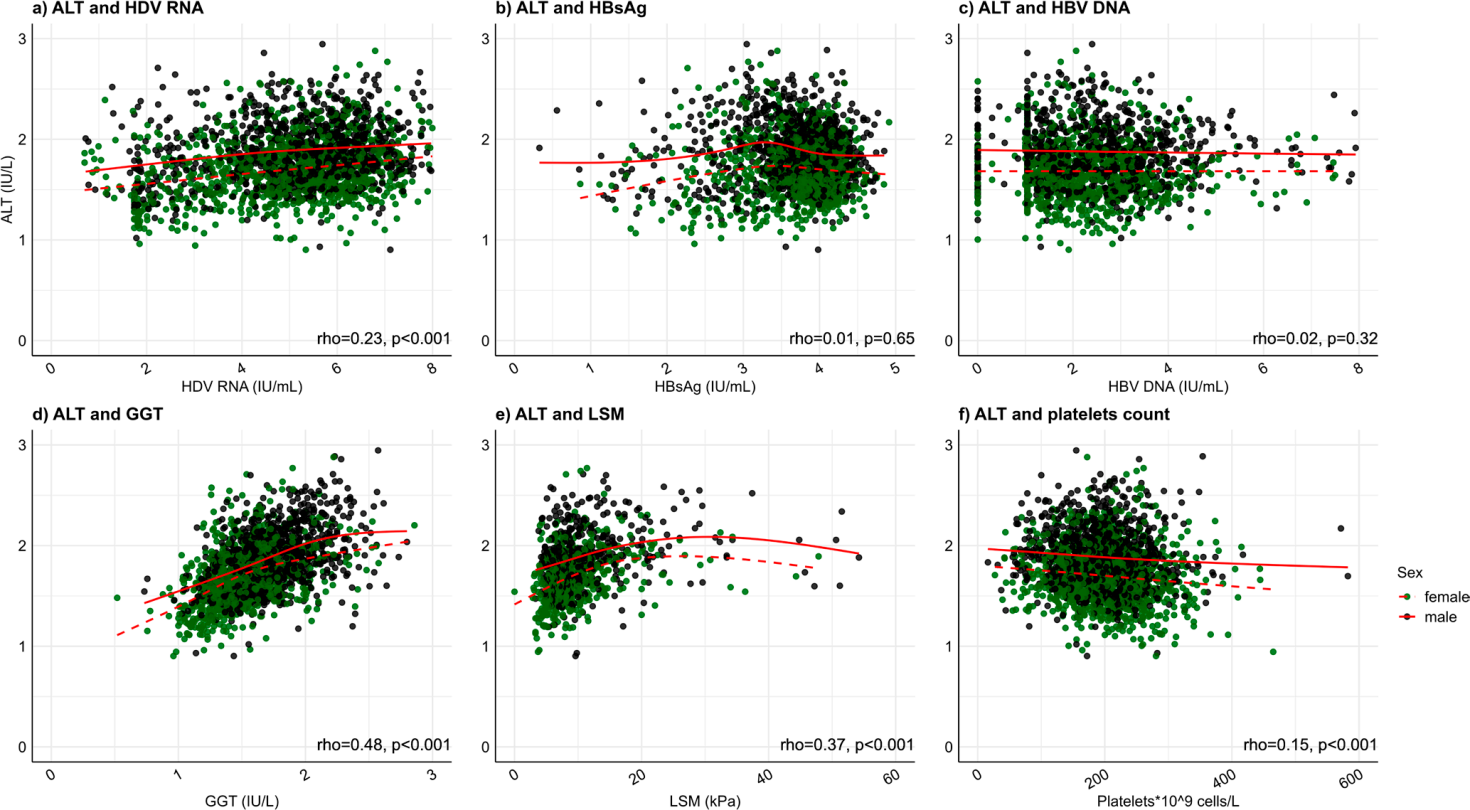
Scatter plots illustrating the association between ALT levels with other parameters in patients with CHD, subgrouped by sex. A flexible regression line (red) illustrates the direction of the association in each group. Spearman correlation coefficients (rho) with *p*‐values are shown for the whole group. Spearman correlation coefficient, rho, *p*‐value. The flexible regression line (red) illustrates the direction of the association. ALT, alanine aminotransferase; Black dots, red line, men, green dots, dashed red line; GGT, gamma glutamyl transferase; HBsAg, hepatitis B surface antigen; HBV DNA, hepatitis B virus deoxyribonucleic acid; HDV RNA, hepatitis D virus ribonucleic acid; LSM, liver stiffness measurement.

As shown in (Figure 3), ALT weakly correlated with HDV RNA (rho = 0.23, *p* < 0.001) and LSM (rho = 0.37, p < 0.001), correlated moderately with GGT (rho = 0.48, *p* < 0.001), and showed a negative weak correlation with platelets count (rho = −0.15, *p* < 0.001). ALT did not correlate with HBV DNA (rho = 0.02, *p* = 0.32). Overall, similar correlations were noted in men and women.

HBsAg showed a very weak positive correlation with HBV DNA levels (rho = 0.07, *p* = 0.004). HBV DNA and HBsAg levels showed negligible or no correlation with platelet counts and LSM levels. Similar correlations were demonstrated when subgrouping by cirrhosis. (Figure S1).

## 4 Discussion

In this cohort study of treatment‐naïve Asian patients with CHD, compared to CHB, we identified following key findings: (1) Younger CHD patients, aged 18–< 30 years, exhibited significantly higher levels of ALT, AST and GGT and more frequent advanced fibrosis than age‐matched CHB patients; (2) Patients with CHD had higher probability of HBeAg+ than propensity‐scored matched CHB. (3) Age < 30 years, elevated GGT and higher HDV RNA levels were significant determinants of elevated ALT in CHD. The present analysis is the largest study to date, examining the prevalence and patterns of ALT elevation in CHD, compared to a matched CHB cohort, offering new insights into the distinct features of HDV infection, with potential implications for HDV care.

Consistent with prior studies, patients with CHD showed higher transaminases and advanced fibrosis compared to CHB, alongside a higher prevalence of HBeAg positivity [6, 21, 22]. Our novel finding of a more frequent and pronounced ALT elevation in young adults with CHD suggests a more active or prolonged phase of immune response targeting infected hepatocytes than previously known compared to HBV‐monoinfection [23]. Earlier studies lacked sufficient young adult data and were not powered for age‐specific analyses [6, 21, 22]. The higher ALT in young adults with CHD may reflect ALT flares related to host immune‐mediated response when transitioning to adulthood; such flares have been described in CHB being more frequent in young adults compared to children or older adults [24]. Later HDV superinfection during adolescence or early adulthood in a high‐endemic region could not be ruled out and agree with our findings of more HBeAg+ in older age groups. The latter hypothesis is consistent with Mongol data estimating HDV prevalence among school children with HBsAg+ at 13.6%, while reaching 60%–80% in adults with HBsAg+, implying that later HDV superinfection is more predominant [25, 26]. Studies suggested a higher HDV infection prevalence among men in rural and eastern Mongol territories, associated significantly with dental procedures and parenteral injections [27].

Unlike CHD with poorly characterised phases in its natural course, the phases of CHB have been defined as follows: HBeAg‐positive chronic HBV infection (immune tolerant phase), HBeAg‐positive chronic hepatitis (immune active phase), HBeAg‐negative chronic HBV infection (inactive carrier phase), HBeAg‐negative chronic hepatitis (immune escape phase), and HBsAg‐negative phase (resolved HBV infection phase) [28]. Individuals acquiring mono‐HBV in adolescence or adulthood often bypass or shorten the “HBeAg+ chronic infection” phase, which typically extends for decades in perinatal or early childhood infected patients [29]. This transition into chronic hepatitis, with a shortened/or without an immune‐tolerant phase, suggests a more accelerated disease course, particularly among older‐age HDV infections [30]. Delayed HBeAg seroconversion, typically occurring at age 30–35 years in HBV‐monoinfection, is associated with increased risk of cirrhosis [31]. In the present analysis, CHD patients aged 30–44 and 40–55 years had 2.0‐ respectively 2.5‐fold higher odds of HBeAg positivity compared to matched CHB peers, indicating delayed seroconversion [32]. Nevertheless, this prevalence of HBeAg+ in middle and older age individuals with CHD in our analysis is higher compared to western cohorts with ~15% HBeAg+ at diagnosis [6, 10]. However, extrapolating HBV phases in CHD remains challenging, with most patients fitting into *HBeAg negative chronic hepatitis* characterised by HBeAg‐, elevated ALT but suppressed HBV DNA levels.

Persistent HDV RNA replication and elevated ALT levels are associated with accelerated risk of cirrhosis and promote carcinogenesis by processes of necroinflammation, fibrosis accumulating oxidative stress, and DNA damage [33]. ALT levels declined in the late stages of advanced fibrosis and cirrhosis, consistent with previous studies, with a negative correlation between ALT and platelets count [33, 34]. However, normal ALT does not exclude ongoing low‐grade hepatic inflammation, also seen in CHB, warranting cautious interpretation of normal ALT levels in CHD [35, 36]. Our findings align with recent EASL and WHO guidelines in considering patients with *detectable* HDV RNA eligible for therapy regardless of age and disease phase, considering the severe necro‐inflammation and fibrosis early in life to prevent later severe liver complications [37, 38]. Similarly, a recent simulated cost‐effectiveness analysis concluded that early treatment (F0 fibrosis of CHD) significantly reduced liver‐related complications, including HCC, and was cost‐effective [39]. Currently, the access of the recently approved anti‐HDV drug bulevirtide is restricted to advanced fibrosis/cirrhosis in some countries with available therapy despite the recommendations [40]. Our findings might motivate amending these recommendations given the accelerated course of CHD; long‐term analyses to confirm our findings are warranted.

ALT normalisation, a surrogate for improved clinical outcomes in CHD under treatment, occurs in 30%–40% with peg‐IFN monotherapy [41], and in 50%–60% with recently introduced bulevirtide monotherapy [42]. In the phase 3 clinical trial of bulevirtide, a discrepancy in the biochemical response of ALT (normalisation of ALT from elevated level at baseline) and the virological response of HDV RNA (≥ 2 log decrease from baseline) was observed during treatment in some patients, with the underlying reason remaining unknown [42]. Patients showing HDV RNA decline but failing to reach ALT normalisation are deemed partial responders. Other confounding factors such as comorbidities of MRF (35% in the current cohort) should be considered when assessing ALT responses [38]. In that context, GGT might be a useful marker of liver disease progression as it correlated with ALT (rho = 0.48, *p* < 0.001), and with cirrhosis agreeing with prior studies, showing the strongest correlation with ALT in the present analysis [43].

We acknowledge several limitations in the present study. The cross‐section design with significant correlations cannot establish causal relationships. Exclusion of patients with anti‐HBV therapies might have enriched the CHB cohort with those with milder disease, despite PSM and similar eligibility criteria applied to both cohorts. Persistent ALT elevation is a stronger determinant of necro‐inflammation and disease progression rather than a single assessment timepoint [44]. Nevertheless, we could note a similar pattern of ALT elevations as baseline when assessed at extended time points. The mode and age at infection were not directly ascertainable; however, HBV in endemic regions is typically acquired perinatally or during early childhood, while a subgroup of HDV superinfection likely occurs later.

Other granular data on HDV or HBV genotype, socioeconomic, alcohol consumption, health behaviours, therapies except for anti‐HBV/HDV therapies were not assessed. Previous studies have though indicated that HDV genotype 1 and HBV genotype D predominate in Mongolia [12]. Hence, our data may not be generalizable to other genotypes. The use of liver stiffness and platelets might misclassify cirrhosis; however, we used recently biopsy‐proven proposed cut‐offs to classify the fibrosis stages [16]. The study setting, reflective of real‐world practice in resource‐limited regions, caused missing data and introduced potential selection bias towards patients with more symptomatic disease and those who can afford tests. Our data include a population of only Asian ethnicity from one country, but the large and rather homogenous cohort in our study could help in understanding ALT elevation and associations in CHD, with statistical power and the possibility to adjust for confounders.

In conclusion, significant liver inflammation, as indicated by elevated ALT levels, higher HBeAg+ distinguishes CHD from CHB in a large cohort of treatment‐naïve patients from Mongolia. Young adults with CHD exhibit a higher inflammatory process, calling for early administration of and more access to anti‐HDV agents. Further studies are needed to examine the long‐term liver‐related outcome in this population and the implication of early treatment on prognosis.

## Author Contributions

All authors contributed to the study conception and design. Material preparation, data collection G.J., S.E., H.K. and analysis were performed by H.K. and D.B. The first draft of the manuscript was written by H.K. and S.A. and all authors commented on previous versions of the manuscript. All authors read and approved the final manuscript.

## Funding

This work was supported by ALF, Region Stockholm, Karolinksa Institutet.

## Ethics Statement

Institutional ethical boards have approved the study; informed consent was waived as the study involves anonymized registers.

## Conflicts of Interest

The authors declare no conflicts of interest.

## Supporting information


**Data S1:** liv70559‐sup‐0001‐Figures.docx.

**Data S2:** liv70559‐sup‐0002‐Tables.docx.

## Data Availability

The data that support the findings of this study are available on request from the corresponding author. The data are not publicly available due to privacy or ethical restrictions.
