# Supplementary material for: Prevalence and Risk Factors of Elevated Alanine Aminotransferase (ALT) in 2382 Treatment‐naïve HBV/HDV Co‐Infected Patients
Source: Liver Int. 2026 Feb 26;46(4):e70559. doi: 10.1111/liv.70559 (PMC12946601; doi:10.1111/liv.70559)
Supplement: Supplementary file 1 — Data S1: liv70559‐sup‐0001‐Figures.docx. [file LIV-46-0-s002.docx]

**sFigure 1:** Scatter dot plots illustrating the association of ALT levels with other parameters in chronic hepatitis D (CHD), subgrouped by cirrhosis*


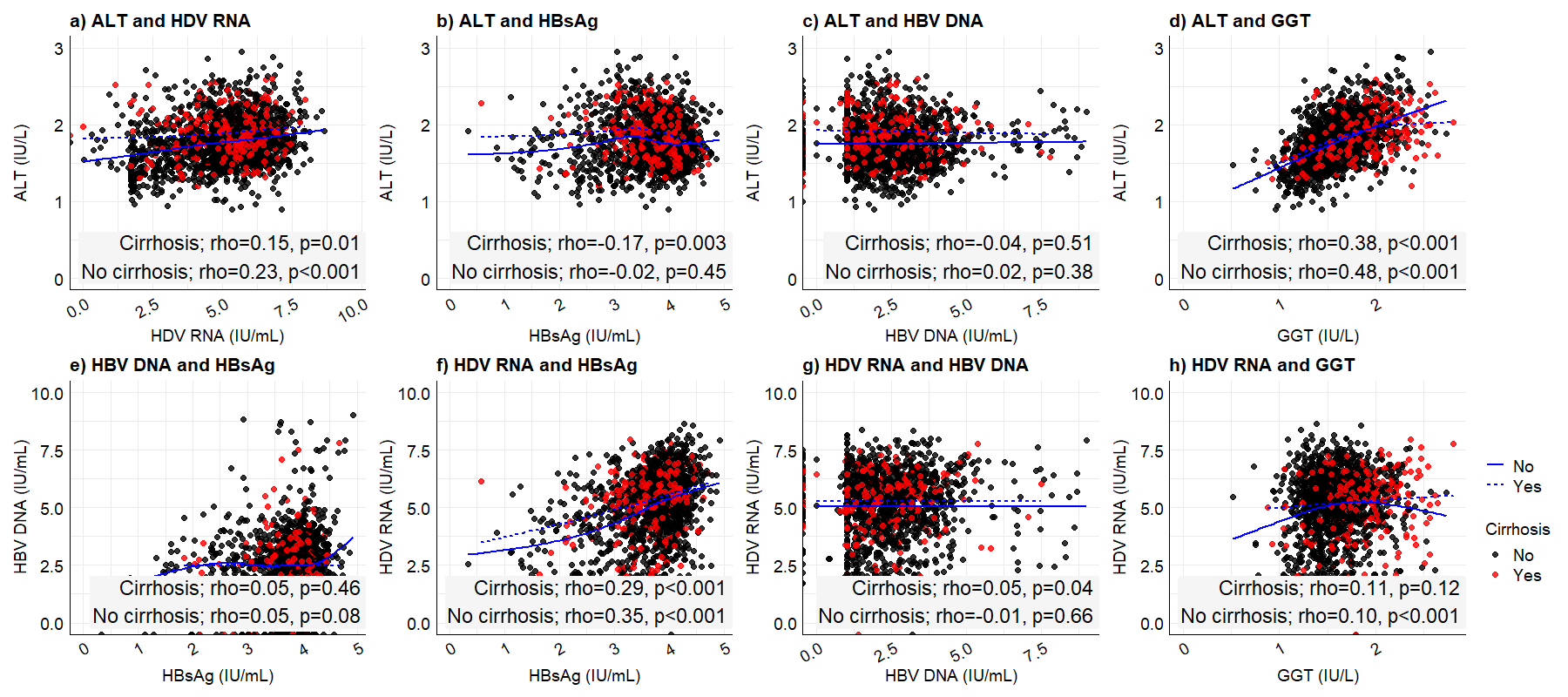


*Cirrhosis is defined as LSM ≥15.2 or platelets count < 150.0*10^9^ cells/L. Spearman correlation coefficient =rho, p-value. The flexible regression line (blue) to illustrate the direction of the association. Abbreviations: ALT=alanine aminotransferase; HDV RNA= hepatitis D virus ribonucleic acid; HBsAg= hepatitis B surface antigen; HBV DNA= hepatitis B virus deoxyribonucleic acid; GGT= gamma glutamyl transferase. Black dots, solid blue line = no cirrhosis, red dots, dashed blue line=cirrhosis.
