# Supplementary material for: Prevalence and Risk Factors of Elevated Alanine Aminotransferase (ALT) in 2382 Treatment‐naïve HBV/HDV Co‐Infected Patients
Source: Liver Int. 2026 Feb 26;46(4):e70559. doi: 10.1111/liv.70559 (PMC12946601; doi:10.1111/liv.70559)
Supplement: Supplementary file 2 — Data S2: liv70559‐sup‐0002‐Tables.docx. [file LIV-46-0-s001.docx]

**Prevalence and risk factors of elevated alanine aminotransferase (ALT) in 2,382** **treatment-naïve HBV/HDV co-infected patients**

Habiba Kamal^1,2^*, Ganbolor Jargalsaikhan^3,4^*, Sanjaasuren Enkhtaivan^3,4^, Karin Lindahl^1,2^, Hannes Hagström^2,6^, Daniel Bruce^5^, Michael Ingre^6^, Bekhbold Dashtserenx^3,4^, Oyungerel Lkhagva-Ochir^3,4^, Tuvshinjargal Ulziibadrakh^3,4^, Andreas Bungert^3,4^, Heiner Wedemeyer^7^, Naranjargal B Dashdorj^3,4#^ and Soo Aleman^1,2#^

Affiliations

1. Department of Infectious Diseases, Karolinska University Hospital, Stockholm, Sweden
2. Department of Medicine Huddinge, Karolinska Institutet, Stockholm, Sweden
3. The Liver Center, Ulaanbaatar, Mongolia
4. ONOM Foundation, Ulaanbaatar, Mongolia
5. Cytel Statistical Consultancy, Stockholm, Sweden
6. Unit of Hepatology, Department of Upper GI Diseases, Karolinska University Hospital, Stockholm, Sweden
7. Department of Gastroenterology, Hepatology, Infectious Diseases and Endocrinology, Hannover Medical School, Hannover, Germany

* shared first authorship, #share senior authorship

| **sTable 1: Baseline characteristics of patients with chronic hepatitis D (CHD) and chronic hepatitis B (CHB) in original study cohort and in propensity scored matched sub-cohorts.** | | | | | | | | | |
| --- | --- | --- | --- | --- | --- | --- | --- | --- | --- |
| **Parameters** | **n=** | **CHD** | **CHB** | **p-value** | **n=** | **All** | **CHD** | **CHB** | **p-value** |
|  | **Main cohort** | | |  | **PSM matched** | | | |  |
| **Number** |  | **2,382** | **1,553** |  |  | **2,742** | **1,371** | **1,371** |  |
| Age at first HBsAg test, years, median (IQR) | 3,935 | 41.2 (34.3, 50.7) | 34.3 (30.1, 41.3) | <0.001 | 2,742 | 35.6 (31.5, 42.6) | 35.9 (31.6, 42.8) | 35.4 (31.4, 42.6) | 0.4 |
| Age <30 years of age | 3,935 | 219 (9.2) | 384 (24.7) | <0.001 | 2,742 | 446 (16.3) | 214 (15.6) | 232 (16.9) | 0.4 |
| **Sex** | 3,935 |  |  | <0.001 | 2,742 |  |  |  | >0.9 |
| Female |  | 1,300 (54.6) | 708 (45.6) |  |  | 1,310 (47.8) | 655 (47.8) | 655 (47.8) |  |
| Male |  | 1,082 (45.4) | 845 (54.4) |  |  | 1,432 (52.2) | 716 (52.2) | 716 (52.2) |  |
| BMI, median (IQR) | 1,468 | 26.4 (23.4, 29.7) | 25.8 (23.2, 29.4) | 0.15 | 1,003 | 26.2 (23.2, 29.5) | 26.3 (23.1, 29.4) | 26.0 (23.6, 29.6) | 0.8 |
| **Laboratory parameters** | | | | | | | | | |
| ALT, IU/L, median (IQR) | 3,935 | 56.6 (36.5, 92.0) | 29.5 (19.6, 48.0) | <0.001 | 2,742 | 42.4 (25.4, 75.3) | 58.5 (38.0, 98.1) | 28.9 (19.4, 47.7) | <0.001 |
| **ALT level categories adjusted for sex** | 3,935 |  |  | <0.001 | 2,742 |  |  |  | <0.001 |
| normal |  | 511 (21.5) | 967 (62.3) |  |  | 1,149 (41.9) | 287 (20.9) | 862 (62.9) |  |
| ≥1-<2 ULN |  | 983 (41.3) | 375 (24.1) |  |  | 882 (32.2) | 555 (40.5) | 327 (23.9) |  |
| ≥2-<5 ULN |  | 721 (30.3) | 170 (10.9) |  |  | 564 (20.6) | 413 (30.1) | 151 (11.0) |  |
| ≥5-<10 ULN |  | 141 (5.9) | 29 (1.9) |  |  | 121 (4.4) | 96 (7.0) | 25 (1.8) |  |
| ≥10 ULN |  | 26 (1.1) | 12 (0.8) |  |  | 26 (0.9) | 20 (1.5) | 6 (0.4) |  |
| AST, IU/L, median (IQR) | 3,925 | 42.1 (30.4, 63.9) | 22.7 (18.1, 32.0) | <0.001 | 2,737 | 30.8 (21.2, 49.1) | 41.5 (30.0, 64.2) | 22.5 (18.0, 31.7) | <0.001 |
| GGT, IU/L, median (IQR) | 2,998 | 39.6 (25.4, 64.8) | 26.6 (18.1, 46.5) | <0.001 | 2,070 | 32.6 (20.8, 55.1) | 38.7 (24.4, 62.9) | 26.8 (18.1, 46.5) | <0.001 |
| Albumin, g/L, median (IQR) | 3,393 | 42.0 (39.6, 44.2) | 44.0 (41.9, 46.0) | <0.001 | 2,359 | 43.2 (41.0, 45.4) | 42.6 (40.2, 44.9) | 43.9 (41.9, 46.0) | <0.001 |
| Total bilirubin, µmol/L, median (IQR) | 3,622 | 12.9 (9.6, 16.8) | 12.0 (9.0, 15.9) | <0.001 | 2,522 | 12.5 (9.3, 16.4) | 13.0 (9.6, 16.9) | 11.9 (9.0, 15.8) | <0.001 |
| Conjugated bilirubin, µmol/L median (IQR) | 2,470 | 4.6 (3.6, 6.1) | 3.9 (3.0, 5.2) | <0.001 | 1,759 | 4.3 (3.2, 5.7) | 4.7 (3.6, 6.3) | 3.9 (2.9, 5.1) | <0.001 |
| Platelets count, 10*9 cells/L, median (IQR) | 3,935 | 199.0 (159.8, 236.0) | 244.0 (208.0, 284.0) | <0.001 | 2,742 | 222.0 (185.5, 263.0) | 204.0 (166.0, 239.0) | 242.0 (207.2, 282.0) | <0.001 |
| **Virological parameters** | | | | | | | | | |
| HBeAg, positive | 972 | 232 (41.4) | 155 (37.6) | 0.2 | 689 | 285 (41.4) | 165 (48.1) | 120 (34.7) | <0.001 |
| qHBsAg log_10_, median (IQR) | 3,045 | 3.8 (3.4, 4.1) | 3.4 (2.8, 3.9) | <0.001 | 2,036 | 3.7 (3.1, 4.0) | 3.9 (3.5, 4.1) | 3.4 (2.7, 3.8) | <0.001 |
| qHBV DNA log_10_, median (IQR) | 2,880 | 2.4 (1.5, 3.2) | 3.4 (2.5, 4.4) | <0.001 | 2,039 | 2.9 (1.9, 3.8) | 2.3 (1.4, 3.2) | 3.4 (2.5, 4.2) | <0.001 |
| qHDV RNA log_10_, median (IQR) | 2,379 | 5.3 (4.3, 6.1) | na | na |  | 4.4 (4.3, 6.3) | 4.4 (4.3, 6.3) | na | na |
| LSM, median (IQRs) earliest visit | 3,935 | 8.3 (6.1, 11.7) | 5.1 (4.2, 6.6) | <0.001 | 2,742 | 6.3 (4.8, 9.1) | 8.1 (6.1, 11.5) | 5.1 (4.2, 6.7) | <0.001 |
| LSM <7.5 kPa | 3,935 | 987 (41.4) | 1,271 (81.8) | <0.001 | 2,742 | 1,704 (62.1) | 585 (42.7) | 1,119 (81.6) | <0.001 |
| LSM ≥10.0 kPa | 3,935 | 835 (35.1) | 127 (8.2) | <0.001 | 2,742 | 581 (21.2) | 469 (34.2) | 112 (8.2) | <0.001 |
| Diabetes | 3,935 | 71 (3.0) | 36 (2.3) | 0.2 | 2,742 | 89 (3.2) | 54 (3.9) | 35 (2.6) | 0.041 |
| MRF | 3,935 | 845 (35.5) | 501 (32.3) | 0.038 | 2,742 | 928 (33.8) | 464 (33.8) | 464 (33.8) | >0.9 |
| Cirrhosis* | 3,935 | 612 (25.7) | 122 (7.9) | <0.001 | 2,742 | 437 (15.9) | 329 (24.0) | 108 (7.9) | <0.001 |
| *Cirrhosis= defined as liver stiffness measurement ≥15.0 for CHD and ≥ 12.5 for CHB or platelets counts <150*10^9^ cells/L.  Number and proportions are presented by column; parameters are presented as n (%) unless stated otherwise. **Abbreviations**: PSM=propensity scored matched; sd: standard deviation; IQR=25^th^ – 75^th^ interquartile; BMI= body mass index; ALT=alanine aminotransferase; AST= aspartate aminotransferase; GGT=gamma glutamyl transferase; ULN= upper limit of normal; LSM=liver stiffness measurements; MRF=metabolic risk factor; na=not applicable. | | | | | | | | | |

| **sTable 2: Parameters associated with CHD diagnosis in propensity scores matched individuals (n=2,742) subgrouped by age. Crude odds ratio (OR) and adjusted (aOR) with 95% confidence interval, CI are presented.** | | | | | | | | | | | | | | | | | |
| --- | --- | --- | --- | --- | --- | --- | --- | --- | --- | --- | --- | --- | --- | --- | --- | --- | --- |
| **Group** | | **All** | | | **18-29** | | | **30-44** | | | **45-59** | | | **≥60** | | | |
| **Univariable model** | | | | | | | | | | | | | | | | | |
| **Predictor** | **Factor** | **OR** | **LCI** | **UCI** | **OR** | **LCI** | **UCI** | **OR** | **LCI** | **UCI** | **OR** | **LCI** | **UCI** | **OR** | **LCI** | **UCI** |  |
| BMI | continuous | 1.00 | 0.97 | 1.02 | 1.02 | 0.94 | 1.10 | 0.99 | 0.95 | 1.02 | 1.01 | 0.95 | 1.08 | 1.04 | 0.90 | 1.22 |  |
| ALT | continuous | 1.01 | 1.01 | 1.01 | 1.02 | 1.02 | 1.03 | 1.01 | 1.01 | 1.01 | 1.01 | 1.01 | 1.02 | 1.00 | 1.00 | 1.01 |  |
| ALT above ULN, IU/L | yes, vs no | 6.40 | 5.40 | 7.59 | 8.20 | 5.28 | 13.04 | 6.55 | 5.30 | 8.12 | 4.94 | 3.34 | 7.39 | 6.40 | 2.49 | 17.64 |  |
| AST above ULN, IU/L | yes, vs no | 1.02 | 1.02 | 1.03 | 11.38 | 7.37 | 17.90 | 7.32 | 5.91 | 9.11 | 5.50 | 3.73 | 8.19 | 7.37 | 2.78 | 21.34 |  |
| GGT above ULN, IU/L | yes, vs no | 7.30 | 6.17 | 8.67 | 2.66 | 1.60 | 4.49 | 1.96 | 1.54 | 2.50 | 2.57 | 1.64 | 4.05 | 0.80 | 0.29 | 2.23 |  |
| Platelet count <150 x 10*9 cells/L | yes, vs no | 6.05 | 4.41 | 8.47 | 4.94 | 1.97 | 15.02 | 8.87 | 5.52 | 15.12 | 6.00 | 3.41 | 11.22 | 0.91 | 0.30 | 2.76 |  |
| HBeAg positive | yes, vs no | 1.75 | 1.29 | 2.37 | 0.91 | 0.46 | 1.77 | 2.03 | 1.38 | 2.99 | 2.51 | 1.04 | 6.40 | 4.20 | 0.40 | 99.96 |  |
| qHBV DNA log_10_ IU/mL | continuous | 0.60 | 0.56 | 0.64 | 0.73 | 0.64 | 0.83 | 0.56 | 0.51 | 0.62 | 0.58 | 0.48 | 0.68 | 0.56 | 0.35 | 0.82 |  |
| qHBV DNA < 2000 IU/mL | yes, vs no | 3.93 | 3.24 | 4.78 | 3.41 | 2.13 | 5.56 | 4.06 | 3.18 | 5.21 | 4.05 | 2.58 | 6.46 | 4.09 | 1.33 | 13.68 |  |
| qHBsAg log_10_IU/mL | continuous | 1.96 | 1.75 | 2.21 | 1.28 | 0.98 | 1.70 | 2.07 | 1.79 | 2.42 | 2.43 | 1.85 | 3.29 | 2.83 | 1.50 | 6.39 |  |
| qHBsAg ≥ 10,000 IU/mL | yes, vs no | 2.82 | 2.31 | 3.47 | 1.57 | 0.98 | 2.55 | 2.96 | 2.31 | 3.81 | 4.38 | 2.43 | 8.28 | 10.06 | 2.39 | 69.95 |  |
| LSM | continuous | 1.26 | 1.23 | 1.30 | 1.37 | 1.26 | 1.49 | 1.35 | 1.30 | 1.41 | 1.15 | 1.11 | 1.21 | 1.07 | 1.00 | 1.16 |  |
| Cirrhosis | yes, vs no | 3.69 | 2.94 | 4.68 | 4.49 | 2.25 | 9.78 | 4.52 | 3.28 | 6.33 | 3.12 | 2.03 | 4.87 | 1.27 | 0.49 | 3.33 |  |
| Diabetes | yes, vs no | 1.57 | 1.02 | 2.43 | 4.47 | 1.10 | 29.81 | 1.56 | 0.91 | 2.72 | 0.96 | 0.39 | 2.37 | 1.90 | 0.18 | 41.88 |  |
| **Multivariable model** | | | | | | | | | | | | | | | | | |
| **Predictor** | **Factor** | **aOR** | **LCI** | **UCI** | **aOR** | **LCI** | **UCI** | **aOR** | **LCI** | **UCI** | **aOR** | **LCI** | **UCI** | **aOR** | **LCI** | **UCI** |  |
| ALT above ULN, IU/L | yes, vs no | 6.11 | 3.72 | 10.2 | 6.53 | 1.84 | 28.40 | 7.21 | 3.99 | 13.50 | 2.12 | 0.53 | 9.07 | 9.60 | 1.85 | 66.90 |  |
| HBeAg positive | yes, vs no | 3.57 | 2.1 | 6.27 | 11.00 | 1.79 | 219.00 | 2.89 | 1.53 | 5.60 | 6.06 | 1.42 | 31.50 |  |  |  |  |
| qHBV DNA < 2000 IU/mL | yes, vs no | 7.95 | 4.74 | 13.8 | 22.30 | 3.56 | 451.20 | 7.09 | 3.85 | 13.70 | 6.13 | 1.65 | 29.01 | 4.36 | 0.83 | 30.60 |  |
| qHBsAg ≥ 10,000 IU/mL | yes, vs no | 1.13 | 0.7 | 1.84 | 0.46 | 0.13 | 1.56 | 1.42 | 0.79 | 2.55 | 2.18 | 0.42 | 12.13 | 3.31 | 0.50 | 30.41 |  |
| Cirrhosis* | yes, vs no | 1.7 | 0.95 | 3.1 | 1.99 | 0.38 | 12.40 | 1.60 | 0.79 | 3.34 | 2.91 | 0.57 | 16.10 |  |  |  |  |
| *Cirrhosis= defined as liver stiffness measurement ≥15.0 for CHD and ≥12.5 for CHB or platelets counts <150*10^9^ cells/L.  Abbreviations: CHD=chronic hepatitis D; BMI= body mass index; ALT=alanine aminotransferase; AST= aspartate aminotransferase; GGT=gamma glutamyl transferase; ULN= upper limit of normal; in men the ULN for ALT was 41 IU/L, was 35 IU/L for AST, was 55 IU/L for GGT and in women 31 IU/L, 31 IU/L and 38 IU/L respectively; LSM=liver stiffness measurements; MRF=metabolic risk factor, Multivariable model adjusted for ALT, HBeAg, HBsAg, HBV DNA, and cirrhosis. | | | | | | | | | | | | | | | | | |
